# Neural encoding of melodic expectations in music across EEG frequency bands

**DOI:** 10.1111/ejn.16581

**Published:** 2024-10-29

**Authors:** Juan‐Daniel Galeano‐Otálvaro, Jordi Martorell, Lars Meyer, Lorenzo Titone

**Affiliations:** ^1^ Max Planck Research Group Language Cycles Max Planck Institute for Human Cognitive and Brain Sciences Leipzig Germany; ^2^ Basque Center on Cognition, Brain and Language (BCBL) Donostia‐San Sebastián Spain; ^3^ Clinic for Phoniatrics and Pedaudiology University Hospital Münster Münster Germany

**Keywords:** cortical tracking, EEG, music, predictions, temporal response function

## Abstract

The human brain tracks regularities in the environment and extrapolates these to predict future events. Prior work on music cognition suggests that low‐frequency (1–8 Hz) brain activity encodes melodic predictions beyond the stimulus acoustics. Building on this work, we aimed to disentangle the frequency‐specific neural dynamics linked to melodic prediction uncertainty (modelled as entropy) and prediction error (modelled as surprisal) for temporal (note onset) and content (note pitch) information. By using multivariate temporal response function (TRF) models, we re‐analysed the electroencephalogram (EEG) from 20 subjects (10 musicians) who listened to Western tonal music. Our results show that melodic expectation metrics improve the EEG reconstruction accuracy in all frequency bands below the gamma range (< 30 Hz). Crucially, we found that entropy contributed more strongly to the reconstruction accuracy enhancement compared to surprisal in all frequency bands. Additionally, we found that the encoding of temporal, but not content, information metrics was not limited to low frequencies, rather it extended to higher frequencies (> 8 Hz). An analysis of the TRF weights revealed that the temporal predictability of a note (entropy of note onset) may be encoded in the delta‐ (1–4 Hz) and beta‐band (12–30 Hz) brain activity prior to the stimulus, suggesting that these frequency bands associate with temporal predictions. Strikingly, we also revealed that melodic expectations selectively enhanced EEG reconstruction accuracy in the beta band for musicians, and in the alpha band (8–12 Hz) for non‐musicians, suggesting that musical expertise influences the neural dynamics underlying predictive processing in music cognition.

## Graphical abstract

We re‐analysed the band‐passed EEG (top right) from subjects who listened to music using multivariate temporal response functions (mTRF). Melodic expectation metrics beyond acoustics (top left) enhanced EEG reconstruction accuracy in all frequency bands below the gamma band (< 30 Hz). Entropy, not surprisal, metrics drove the effect in all bands (bottom left). The enhancement was stronger in the beta band (12–30 Hz) for musicians and in the alpha band (8–12 Hz) for non‐musicians (bottom right).

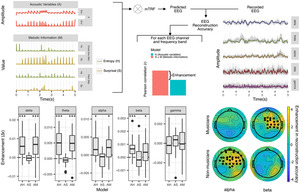

**Abbreviations**
A: Baseline TRF model trained solely on Acoustic (A) variables
AF: TRF model trained on Acoustic (A) and one (or more) melodic information Feature(s) (F)
AIC: Akaike information criterion
AM: Omnibus TRF model trained on Acoustic (A) and all Melodic information (M) variables
EEG: Electroencephalography
H: Note entropy
IDyOM: Information Dynamics Of Music
TRF: Temporal Response Function
O: Note onset time
P: Note pitch
S: Note surprisal

## 1 INTRODUCTION

Speech and music contain multiple sorts of regularities, which the brain can exploit to predict future events (Arnal & Giraud, 2012; Friston, 2005; Rimmele et al., 2018). Prior work showed that low‐frequency brain activity synchronizes with the temporal structure of rhythmic events to optimize sensory processing (Giraud & Poeppel, 2012; Haegens & Zion Golumbic, 2018; Henry & Obleser, 2012; Lakatos et al., 2008; Meyer, 2018; Meyer et al., 2020). Yet, the neural dynamics of predictive processing may also involve brain activity at higher frequencies (Arnal & Giraud, 2012; Bastos et al., 2012).

Research in auditory neuroscience revealed that delta‐band (< 4 Hz) oscillatory phase aligns to the timing of predicted events (Arnal et al., 2015; Breska & Deouell, 2017; Herbst et al., 2022; Stefanics et al., 2010; Tal et al., 2017). Low‐frequency phase alignment has often been studied within a cortical tracking framework, which relates different timescales of brain activity to distinct stimulus features (e.g., Giraud & Poeppel, 2012; Keitel et al., 2018). Yet, next to tracking dynamics at low frequencies, high‐frequency power dynamics in the beta (13–30 Hz) and gamma band (> 30 Hz) have been associated with predictions and prediction errors, respectively (Armeni et al., 2019; Bastos et al., 2012; Engel & Fries, 2010; Herrmann et al., 2004; Omigie et al., 2019; Sankaran et al., 2024; van Pelt et al., 2016; Wang et al., 2012). Several studies also reported that beta‐band power couples to delta‐band phase prior to temporally expected events (Arnal et al., 2015; Chang et al., 2019; Cravo et al., 2011; Morillon & Baillet, 2017; Saleh et al., 2010; Zalta et al., 2024), and that it “rebounds” after prediction errors (Armeni et al., 2019; Arnal et al., 2011; Arnal & Giraud, 2012; Basanisi et al., 2023; Doelling & Poeppel, 2015; Engel & Fries, 2010; Fujioka et al., 2012; Palmer et al., 2019). Taken together, these studies suggest the joint involvement of neural dynamics at low and high frequencies in predictive processing. Moreover, temporal and content predictions have recently been proposed to share the same neurophysiological substrate via oscillatory phase coding (Ten Oever & Martin, 2021, 2024). According to this framework, low‐frequency neural activity arranges content predictions along a temporal gradient of predictability, so that more likely representations may reach activation at earlier, more excitable, oscillatory phases.

Cognitive models of predictive processing postulate that the brain predicts events given a context and updates its prior probabilities based on prediction errors (Arnal & Giraud, 2012; Bastos et al., 2012). Predictions and prediction errors can be computationally modelled with two information‐theoretical metrics: entropy, a measure of uncertainty surrounding the predictions, that tells us how hard it is to make a prediction of the next event; and surprisal, a proxy of prediction error, that tells how unlikely a stimulus is (Shannon, 1948). These metrics can also be adapted to describe the temporal and content predictability of an event, separately (Di Liberto, Pelofi, Bianco, et al., 2020; Pearce, 2018).

Multivariate approaches that use linear regression models, such as temporal response functions (TRF; Crosse et al., 2016, 2021) have been employed to study the neural encoding of surprisal and entropy features in both speech (Gillis et al., 2021; Weissbart et al., 2020) and music (Di Liberto, Pelofi, Bianco, et al., 2020; Kern et al., 2022; Sankaran et al., 2024). In line with the cortical tracking framework, these approaches mostly focused on low‐frequency (1–8 Hz) dynamics, typically comprising broadband neural activity. For instance, Di Liberto, Pelofi, Bianco, et al. (2020)’s findings that note surprisal and note entropy enhance broadband low‐frequency EEG reconstruction accuracy beyond acoustic features and that these melodic expectation features are encoded at different latencies can be taken as evidence for cortical tracking of changes in the stimulus properties along these dimensions. Nevertheless, TRFs can also be modelled to predict EEG in narrower frequency ranges separately, allowing for the study of how melodic expectations are encoded across distinct frequency bands beyond broadband low‐frequency dynamics. In this context, encoding of melodic expectations in high‐frequency bands (e.g., > 60 Hz) has been found in intracranial research (Omigie et al., 2019; Sankaran et al., 2024). However, it remains unclear whether distinct frequency bands selectively encode temporal and content predictions and prediction errors in music.

**FIGURE 1:**
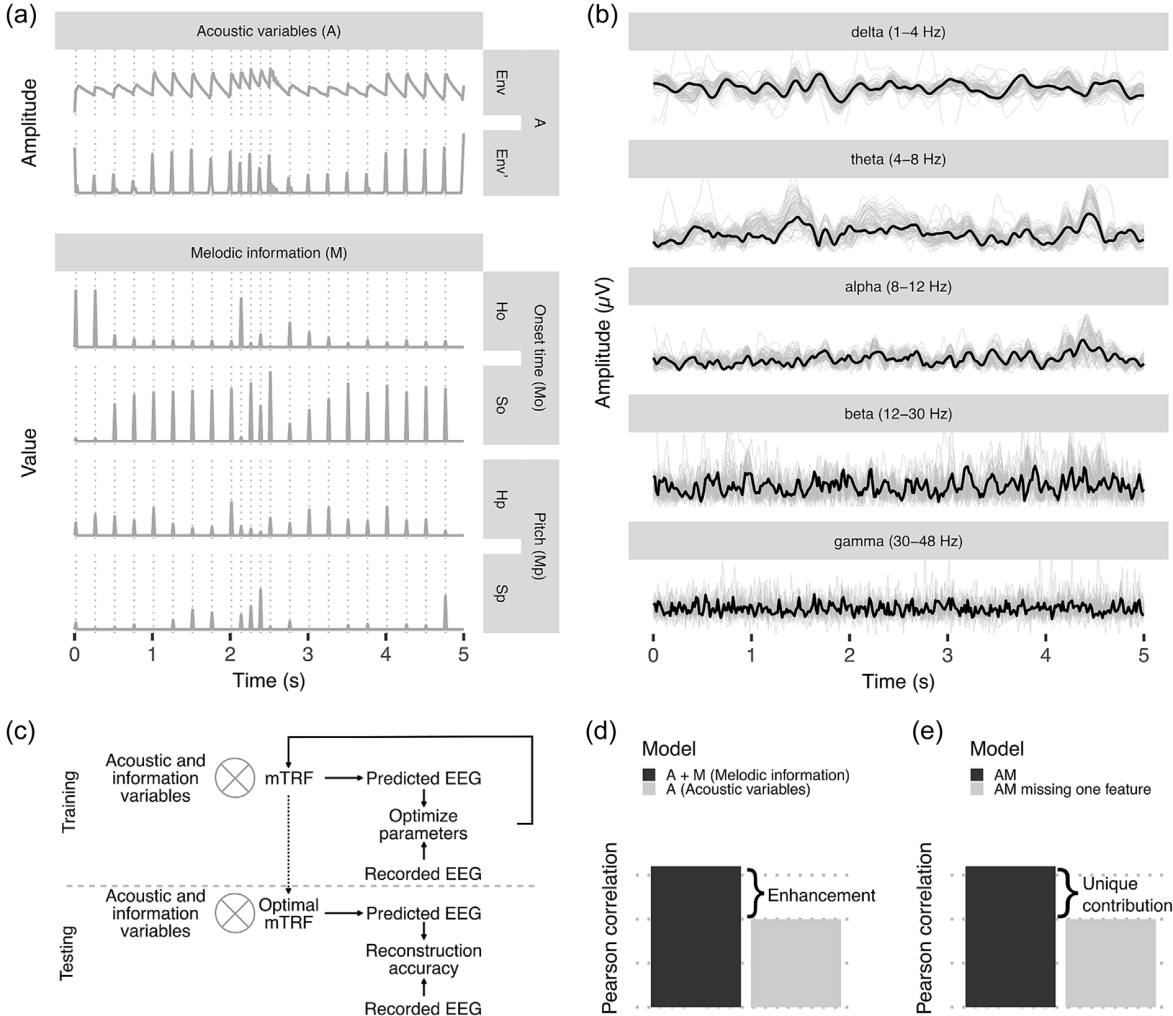
Overview of the study. A, Snippet of the sets of regressors obtained from one piece of music used to calculate the TRFs. From top to bottom: acoustic envelope (Env), half‐way rectified first derivative of the envelope (Env’); melodic information for note onset (M_o_): onset entropy (H_o_), onset surprisal (S_o_); melodic information for note pitch (M_p_): pitch entropy (H_p_), and pitch surprisal (S_p_). Based on Di Liberto, Pelofi, Bianco, et al. (2020). B, Snippet of pre‐processed EEG bands of a single subject used for TRF analysis. Gray lines represent individual channels and black lines represent the average amplitude across electrodes for each frequency band. C, Schematic of the pipeline. Melodic information features were extracted with IDyOM (Di Liberto et al., 2021; Pearce, 2005). Separate multivariate temporal response function (mTRF) models were trained from the preprocessed EEG signal and the acoustic and melodic information features (see Materials and methods). The sets of features were convolved with the corresponding optimal TRFs to predict the EEG signals. The reconstruction accuracy is calculated as the Pearson correlation between the original filtered EEG and the predicted one. D, Hypothetical enhancement in EEG reconstruction accuracy of a model trained on acoustic and melodic information variables (AM) compared to a model trained only on acoustic regressors. Based on Di Liberto, Pelofi, Bianco, et al. (2020). E, Hypothetical unique contribution of an individual feature to the full model, measured as the difference in the Pearson correlation between the reconstruction accuracy obtained using the AM model and the same model missing one specific feature.

In this study, we re‐analysed the dataset from Di Liberto et al. (2021) and investigated the EEG correlates of prediction uncertainty (entropy) and prediction error (surprisal) in music across different frequency bands. To this end, we trained several TRF models and regressed temporal (note onset) and content (note pitch) entropy and surprisal metrics on narrow‐band EEG data, while factoring out acoustic confounds (Figure 1). Taking into account the proposed neural mechanisms behind predictions and prediction error described above, we expected delta‐ and beta‐band activity to encode temporal uncertainty surrounding predictions (onset entropy) before stimulus onset (Arnal et al., 2015; Bastos et al., 2012; Fujioka et al., 2012; Hansen & Pearce, 2014); and beta‐ and gamma‐band activity to encode prediction errors (note surprisal) after stimulus presentation (Engel & Fries, 2010; van Pelt et al., 2016; Wang et al., 2012). Building on previous evidence that musical expertise enhances the processing of music (Di Liberto, Pelofi, Shamma, & de Cheveigné, 2020; Doelling & Poeppel, 2015; Quiroga‐Martinez et al., 2020b), we also explore to what extent musicians' (versus non‐musicians') brain responses display enhanced encoding of entropy and surprisal in distinct frequency bands.

## 2 MATERIALS AND METHODS

### 2.1 Dataset

The dataset was obtained from Di Liberto et al. (2021). It contained EEG recordings from 20 healthy subjects (10 females, mean age = 29), half of which were professional pianists. The stimuli consisted of 10 monophonic piano melodies (~150 s each) extracted from works of Johann Sebastian Bach. Subjects listened to each piano melody three times for a total of 30 trials, while their EEG data were continuously recorded with a 64‐electrode cap and digitized at 512 Hz with a Biosemi Active Two system (Di Liberto, Pelofi, Bianco, et al., 2020).

The acoustic‐related vectors provided by Di Liberto et al. (2021) encompassed the note onset vector, the note pitch vector and the music envelope. The note onset vector consisted of a binary vector of the length of the musical piece with ones at the start of each note and zeros elsewhere. The note pitch vector had the MIDI value of each note at its onset position and zeros elsewhere. As in the original study (Di Liberto, Pelofi, Bianco, et al., 2020), the music envelope was computed as the absolute value of the Hilbert transform of the music sound wave, and its derivative was computed as the half‐way rectified first‐order derivative of the music envelope. These regressors were used to control for the covariance between the acoustic properties of the stimulus and the variables associated with the cognitive processes of interest (i.e., prediction and prediction errors), operationalized by entropy and surprisal (Crosse et al., 2021).

Melodic surprisal and entropy were calculated using the Information Dynamics of Music (IDyOM), a computational model pre‐trained on Western music that uses statistical learning to calculate information‐theoretical metrics, such as the likelihood of an event in a particular context (Pearce, 2005, 2018). Surprisal ( S) was calculated as in Equation 1, where $p\left(e_{i}|e_{1..i-1}\right)$ is the probability of the note $e_{i}$ given the note sequence $e_{1..i-1}$. Thus, it represents the inverse probability of a note given its previous context in a melody. Unexpected notes in a very specific context elicit high surprisal values, and vice versa. Entropy ( H) was computed as in Equation 2, and defined mathematically as the average of surprisal over all possible continuations in a sequence (Shannon, 1948). Thus, it reflects a measure of uncertainty. More uncertain (less predictable) continuations lead to higher entropy values, and vice versa. Surprisal and entropy were computed separately for note onset and pitch (H_o_, H_p_, S_o_, S_p_) to enable the investigation of the neural encoding of temporal and content predictions and prediction errors in different EEG frequency bands. Note entropy and surprisal were encoded along the time dimension by assigning their value to the corresponding time point in the note onset vector (Figure 1A).

(1) $$S\left(e_{i}|e_{1..i-1}\right)=\log_{2}\frac{1}{p\left(e_{i}|e_{1..i-1}\right)}$$

(2) $$\begin{array}{c} H\left(e_{1..i-1}\right)=\sum_{e\in E}p\left(e|e_{1..i-1}\right)S\left(e|e_{1..i-1}\right) \end{array}$$

### 2.2 EEG preprocessing

The EEG recordings were preprocessed using MATLAB (version R2022b), the FieldTrip toolbox (Oostenveld et al., 2011) and the EEGLAB toolbox (Delorme & Makeig, 2004). The broad‐band EEG was digitally filtered with two‐pass second‐order Butterworth filters, and split into five frequency bands (Figure 1B): delta (1–4 Hz), theta (4–8 Hz), alpha (8–12 Hz), beta (12–30 Hz) and gamma (30–48 Hz). A low‐frequency band (1–8 Hz) was kept to replicate the frequency range used by Di Liberto, Pelofi, Bianco, et al. (2020). EEG channels exceeding three times the mean standard deviation across all other channels were removed and replaced with spline interpolation. Next, all channels were re‐referenced to the mean of the two mastoid channels. For all frequency bands except for delta, we calculated the instantaneous power modulation as the absolute value of the Hilbert transform (Teoh et al., 2019; Weissbart et al., 2020). This transformation aids the interpretation of TRFs in higher frequency bands as it will lead to single peaks at a time *t* instead of the response activity being spread around this point with negative and positive peaks (Etard et al., 2019; Van Canneyt et al., 2021). As delta band corresponds to low‐frequency activity, the resulting TRF can be interpreted more easily without further transformations (Weissbart et al., 2020). Frequencies below .5 Hz and above 20 Hz were filtered out of the result with second‐order Butterworth filters as they are not relevant for the analyses. The resulting EEG was down‐sampled to 64 Hz.

### 2.3 Temporal response function (TRF)

Temporal response function (TRF) models were calculated using the mTRF toolbox (Crosse et al., 2016). The TRF is the set of weights obtained as a regression of the features from the stimuli and the EEG data. They solve Equation 3, where *r* is the instantaneous neural response at time *t* and channel *n*; w represents the weight for that channel and time point; *s* represents a stimulus feature; and ϵ the error to be minimized (Crosse et al., 2016). Additionally, the value τ represents a range of time lags to be taken into account due to the non‐instantaneous nature of the neural response (Crosse et al., 2016). For instance, Di Liberto, Pelofi, Bianco, et al. (2020), used an initial window of −150 to 750 ms, but proceeded to narrow it to 0 to 350 ms to reduce overfitting. Since, we were interested in the neural response before and after the onset of stimuli, we calculated the TRFs in the original time window (−150 to 750 ms).

(3) $$\begin{array}{c} r\left(t,n\right)=w\left(\tau ,n\right)s\left(t-\tau \right)+\epsilon \left(t,n\right)\; \end{array}$$

The TRF weights (*w*) were calculated for each regressor as in Equation 4, where **S** is a lagged time series of the stimulus features, **r** is the neural response, λ is a regularization factor and **M** is a matrix that depends on the regularization method. We used a ridge regularization method, where **M** is simply the identity matrix, as it prevents possible cross‐channel leakage for multivariate input features (Crosse et al., 2016, 2016; Wong et al., 2018). The calculation of the TRF weights was performed using a nested leave‐one‐out cross‐validation, where each trial was left‐out once as a test‐fold and the rest were used for an internal cross‐validation loop to estimate the optimal λ regularization parameter (Crosse et al., 2016). Thus, each trial was kept for validation once while the weights were calculated using the training trials and a λ chosen from the range 10^−6^ to 10^6^. The best λ was chosen as the one that maximized the reconstruction accuracy, which is defined as the average Pearson correlation across channels between the validation trial and the predicted EEG. So, we obtained one set of TRF weights per trial with the optimal λ, and one value of reconstruction accuracy per channel (Figure 1C).

(4) $$\begin{array}{c} w={\left(S^{T}S+\lambda M\right)}^{-1}-S^{T}r\; \end{array}$$

To test how well onset and pitch surprisal and entropy metrics predict the EEG signal beyond acoustic features, we trained TRF models with different sets of regressors (Figure 1A). The baseline model (A) was trained with acoustic features. The full model (AM) was trained with all melodic information (M) and acoustics (A) features. To assess the unique contribution of individual features to the EEG reconstruction accuracy, we trained TRF models with one or more information features (AF) and models missing one of the features (AM ‐ F). Lastly, we calculated a baseline model with preserved acoustic information, but with shuffled non‐zero surprisal and entropy values (AM‐shuffled) to account for differences in the dimensionality between AM and A (Crosse et al., 2021; Di Liberto, Pelofi, Bianco, et al., 2020; Weissbart et al., 2020).

### 2.4 Statistical analyses

To assess if entropy and surprisal features for note onset and pitch improve the reconstruction accuracy beyond acoustic features, we compared the EEG reconstruction accuracy of different TRF models using one‐sided Wilcoxon signed‐rank tests (Gillis et al., 2021). For each EEG frequency band, we first compared the reconstruction accuracy of the AF models versus the A model to assess the added value of each feature beyond the acoustic baseline (Figure 1D), which is referred to as “enhancement”. Next, we compared the reconstruction accuracy of TRF models trained on all but one feature (AM – F) versus the AM model (Figure 1E). The logic here is that a loss in reconstruction accuracy for the AM – F model indicates that the left‐out feature contributes to explain unique variance while controlling for the presence of the other features. Therefore, we refer to this value as “unique contribution” of individual features to the model above and beyond other melodic information variables. Importantly, this approach has the advantage of controlling for collinearity. The analysis of EEG reconstruction accuracy contrasting more complex TRF models (AF) versus a baseline model (A) is arguably the most common approach adopted in previous studies using mTRF (Crosse et al., 2021; Di Liberto, Pelofi, Bianco, et al., 2020; Gillis et al., 2021; Kern et al., 2022). On the one hand, the enhancement metric can be taken as a sort of sanity check or confirmatory analysis of the unique contribution metric with respect to previous TRF studies. On the other hand, the analysis of enhancement in AF models also allows us to assess differences in reconstruction accuracy among models trained on different combinations of features (onset vs. pitch, surprisal vs. entropy). However, the unique contribution approach better quantifies the encoding of each individual feature than the enhancement, since it controls for the contribution of other features to the performance of the model. Hence, both approaches provide complementary evidence for our research interests: (i) the enhancement in reconstruction accuracy metric allows us to compare models with multiple features to isolate the effects of sets of regressors (e.g., entropy for both time and pitch); (ii) the unique contribution metric allows us to isolate the effect of individual regressors. In the tables, we report the effect sizes calculated as the z‐scores divided by the number of observations (Field et al., 2012). Effect sizes greater than .5 indicate a large effect (Cohen, 1992). To assess if some electrodes show an enhancement in reconstruction accuracy between the AM and A models, we performed mass univariate cluster‐based permutation tests (Maris & Oostenveld, 2007) with 10,000 permutations of each trial for every subject, and a significance threshold of .05 (Gillis et al., 2021). The comparison between the AM model and the AM‐shuffled model was calculated using 1,000 permutations of each trial for every subject.

EEG channels that showed a significant reconstruction accuracy enhancement (cluster‐based permutation tests) within each frequency band were selected to examine the predicted neural response of the subjects. The predicted EEG response was tested by analysing the consistency of the TRF amplitude peaks across subjects (TRF weights). The TRF weights of each regressor have a dimensionality of N × T, where N is the number of channels and T is the number of lags analysed. To reduce inter‐subject peak latency variability we applied a 100 ms smoothing Hamming kernel (Gillis et al., 2021). The latency of the peaks was defined as the time of maximum absolute amplitude in each window of time (Gillis et al., 2021). To extract the latency in which the TRF weights were consistent across subjects, we tested whether the weights were significantly different from zero with permutation tests by randomly flipping sign to the TRF weights and applying threshold‐free cluster enhancement (Smith & Nichols, 2009). Differences in peak latencies within each frequency band were calculated using two‐tailed Wilcoxon sign‐rank tests.

To assess the interaction between melodic information features and frequency bands on the reconstruction accuracy enhancement, we fitted different linear mixed‐effects models using the results for the AH_o_, AH_p_, AS_o_ and AS_p_ models. Linear mixed‐effects models were estimated using maximum likelihood and compared with likelihood ratio tests using R and the lme4 package (Bates et al., 2015). The analysis of the main effects within each model was performed using a type III ANOVA. We defined a null model included only a categorical term for FREQUENCY (delta, theta, alpha, beta) as a fixed effect, and alternative models including a term to account for either FEATURE (H_o_, H_p_, S_o_, S_p_), kind of METRIC (entropy, surprisal) or feature TYPE (note pitch, note onset), and their interaction with FREQUENCY (FEATURE × FREQUENCY, METRIC × FREQUENCY or TYPE × FREQUENCY). In addition, the effect of musical expertise was analysed by comparing the performance of the AM models between musicians and non‐musicians. Based on the EEG reconstruction accuracy findings, we fitted a null model including a fixed factor for FREQUENCY (alpha, beta), and alternative models including an additional fixed factor for EXPERTISE (musician or non‐musician) and for the EXPERTISE × FREQUENCY interaction.

## 3 RESULTS

### 3.1 EEG reconstruction accuracy

**FIGURE 2:**
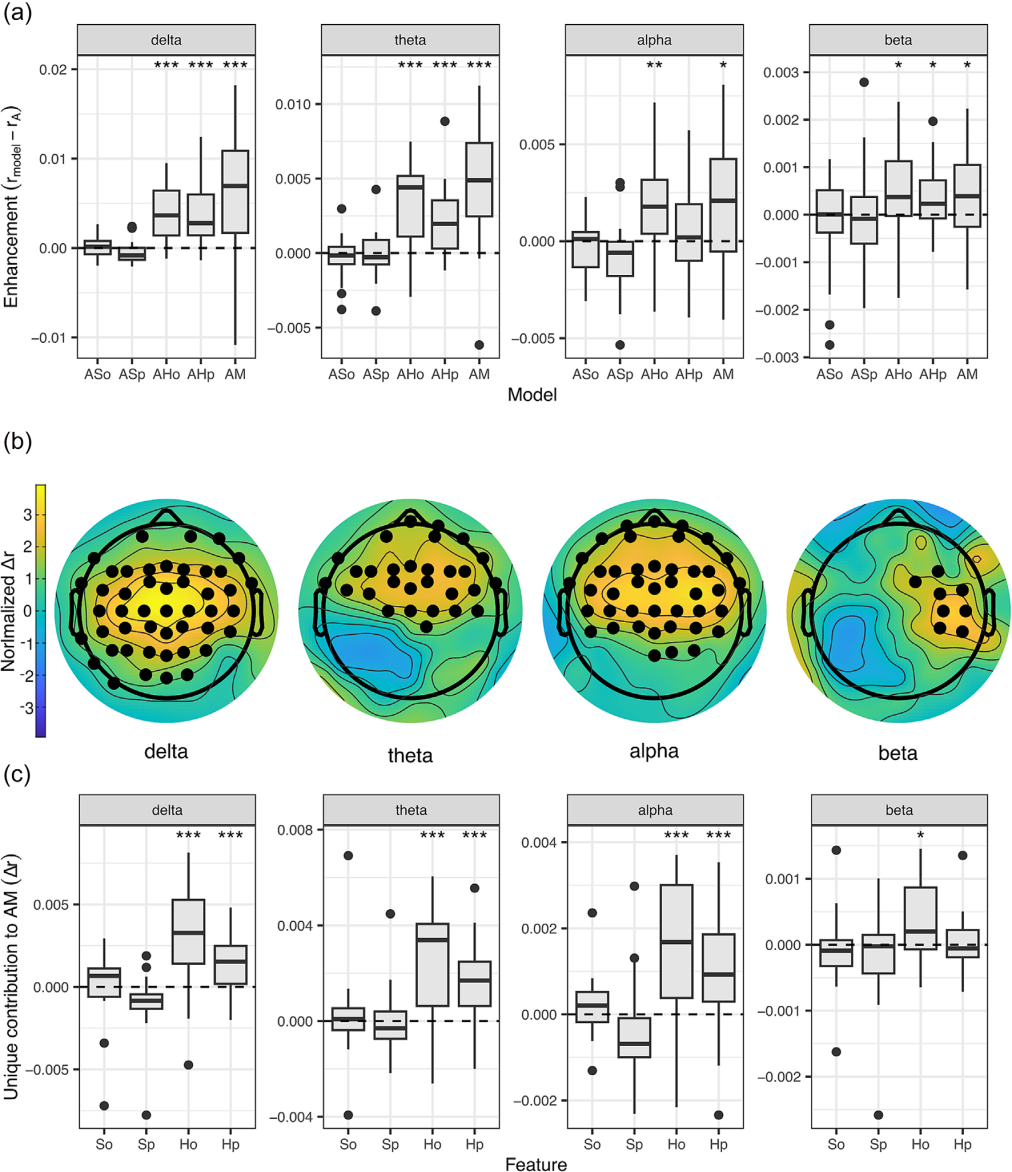
Frequency‐specific EEG reconstruction accuracy enhancement. A, Enhancement in EEG reconstruction accuracy for models trained with an additional information feature versus the baseline model trained with only acoustic features (Wilcoxon sign‐rank test; *p < .05, **p < .01, ***p < .001). Abbreviations as in Figure 1A. B, Electrodes with a significant EEG reconstruction accuracy enhancement for the AM model versus the A model (cluster‐based permutation test; 10,000 permutations; *p* < .05). C, Unique contribution of each individual feature to the EEG reconstruction accuracy of the full AM model, measured as the difference in EEG reconstruction accuracy between the full model and the model missing one feature (Wilcoxon sign‐rank test; *p < .05, **p < .01, ***p < .001). Black dots represent outlier values.

The contribution of surprisal and entropy regressors for note onset and pitch to the narrow‐band EEG reconstruction accuracy was tested beyond the contribution of acoustic regressors. This analysis revealed a significant reconstruction accuracy enhancement for all frequency bands, except for the gamma band (Figure 2A; Table 1). Hence, the gamma band was omitted from further analyses. The performance of the AM model versus the AM‐shuffled model confirmed the enhancement in EEG reconstruction accuracy for all frequency bands (delta: *p* < .001, effect size = .877; theta: *p* < .001, effect size = .869; alpha: *p *< .001, effect size = .776; beta: *p* = .009, effect size = .526). TRF models trained with individual features beyond the acoustic showed different results in distinct frequency bands (Figure 2A, Table 1). More specifically, AH_o_ and AH_p_ showed a significant enhancement in the delta‐, theta‐ and beta‐band compared to the baseline model; while in the alpha band, only AH_o_ showed a significant enhancement. To investigate which electrodes showed a significant enhancement for the AM model compared to the baseline A, we performed cluster‐based permutation tests. These tests showed that delta‐, theta‐ and alpha‐band reconstruction accuracy was enhanced over fronto‐central electrodes, while the effect in the beta band seemed distributed over the right hemisphere (Figure 2B). Moreover, we tested which regressors had a unique contribution to the AM model (Figure 2C, Table 2). This analysis revealed that only H_o_ showed a unique contribution in the beta band, while both entropy regressors (H_o_ and H_p_) had a unique contribution in the other bands. Finally, we compared models including combined regressors for entropy (AH) and surprisal (AS) and for note onset (AM_o_) and note pitch (AM_p_) features (Figure S1, Table 3). Here, AH and AM_o_ showed an enhancement in reconstruction accuracy for all bands; while the enhancement for AM_p_ was limited to the delta and theta bands. Based on this result, we analysed the unique contribution of H_o_ and H_p_ to the AH model. This test revealed a significant contribution of both H_o_ and H_p_ for all frequency bands (Figure S2, Table 4). In sum, entropy regressors, especially for note onset (H_o_), led to a significant enhancement in all bands, while surprisal regressors did not.

**TABLE 1:** Reconstruction accuracy enhancement (AF – A).

Model	AM		AH_o_		AH_p_		AS_o_		AS_p_	
Frequency	*p* value	Effect size	*p* value	Effect size	*p* value	Effect size	*p* value	Effect size	*p* value	Effect size
Delta	<.001	.706	<.001	.843	<.001	.776	.249	.159	.938	.342
Theta	<.001	.760	<.001	.734	<.001	.718	.774	.167	.702	.116
Alpha	.012	.497	.002	.634	.311	.116	.774	.141	.702	.467
Beta	.016	.476	<.001	.476	.016	.476	.594	.050	.702	.467
Gamma	.324	.108	.285	.134	.912	.300	.435	.042	.298	.125

**TABLE 2:** Unique contribution of each feature (AM – AF).

Feature	H_o_		H_p_		S_o_		S_p_	
Frequency	*p* value	Effect size	*p* value	Effect size	*p* value	Effect size	*p* value	Effect size
Delta	<.001	.718	.001	.651	.108	.283	.997	.584
Theta	<.001	.742	<.001	.726	.311	.116	.739	.142
Alpha	<.001	.476	<.001	.659	.108	.283	.943	.350
Beta	.016	.476	.507	.000	.869	.250	.826	.208

**TABLE 3:** Reconstruction accuracy enhancement for sets of features (AF – A).

Model	AM_o_		AM_p_		AH		AS	
Frequency	*p* value	Effect size	*p* value	Effect size	*p* value	Effect size	*p* value	Effect size
Delta	<.001	.776	<.001	.726	<.001	.784	.727	.133
Theta	<.001	.734	.003	.601	<.001	.776	.899	.283
Alpha	.024	.442	.608	.058	.004	.576	.971	.417
Beta	.002	.609	.165	.225	<.001	.726	.622	.066

**TABLE 4:** Unique contribution of each entropy feature (AH – AF).

Feature	H_o_		H_p_	
Frequency	*p* value	Effect size	*p* value	Effect size
Delta	<.001	.684	.001	.651
Theta	<.001	.726	.002	.636
Alpha	<.001	.701	.165	.225
Beta	.013	.492	.027	.434

To further assess the interaction between features and frequency bands, we fitted linear mixed‐effects models and ran model comparisons. We conducted an ANOVA test to compare a null model with FREQUENCY (delta, theta, alpha, beta) as a fixed factor and an alternative model that additionally included FEATURE (H_o_, H_p_, S_o_, S_p_). This test revealed that FEATURE significantly contributed to explaining reconstruction accuracy (Akaike information criterion, AIC = −3018.7, *χ* ^2^(3) = 89.20, *p *< 10^−15^) relative to the null model (AIC = −2935.5). Moreover, the interaction FREQUENCY x FEATURE revealed a contribution above the previous model (AIC = −3038.0, *χ* ^2^(9) = 37.31, *p *< .001), suggesting differential feature encoding depending on the frequency bands. Post‐hoc pairwise comparisons confirmed that entropy regressors provided a greater contribution in the delta and theta bands than in the alpha and beta bands (*p *< .05, Tukey's method for multiple comparisons). On the other hand, surprisal regressors did not significantly improve reconstruction accuracy between frequency bands (*p *> .05). We also considered the influence of the kind of METRIC (entropy, surprisal) and feature TYPE (note pitch, onset time) on the reconstruction accuracy in different frequency bands. A comparison between a null model with only FREQUENCY as a fixed factor versus a model that also included METRIC showed that the latter significantly improved the model performance (AIC = −3017.3, *χ* ^2^(1) = 83.79, *p* < 10^−15^). Moreover, the interaction METRIC x FREQUENCY further improved the model performance (AIC = −3043.9, *χ* ^2^(3) = 32.62, *p *< 10^−6^). Pairwise comparisons confirmed that the effect of entropy was higher than the effect of surprisal in enhancing the reconstruction accuracy in all bands, except beta (delta: *t*(293) = 8.927, *p* < .001; theta: *t*(293) = 6.534, *p* < .001; alpha: *t*(293) = 3.897, *p* < .001; beta: *t*(293) = 1.611, *p* = .287). Conversely, the factor TYPE showed only a small improvement relative to the null model (AIC = −2936.4, *χ* ^2^(1) = 2.98, *p *= .084), with no further improvement for the FREQUENCY x TYPE interaction (AIC = −2932.1, *χ* ^2^(3) = 1.66, *p *= .645).

### 3.2 TRFs weights

**FIGURE 3:**
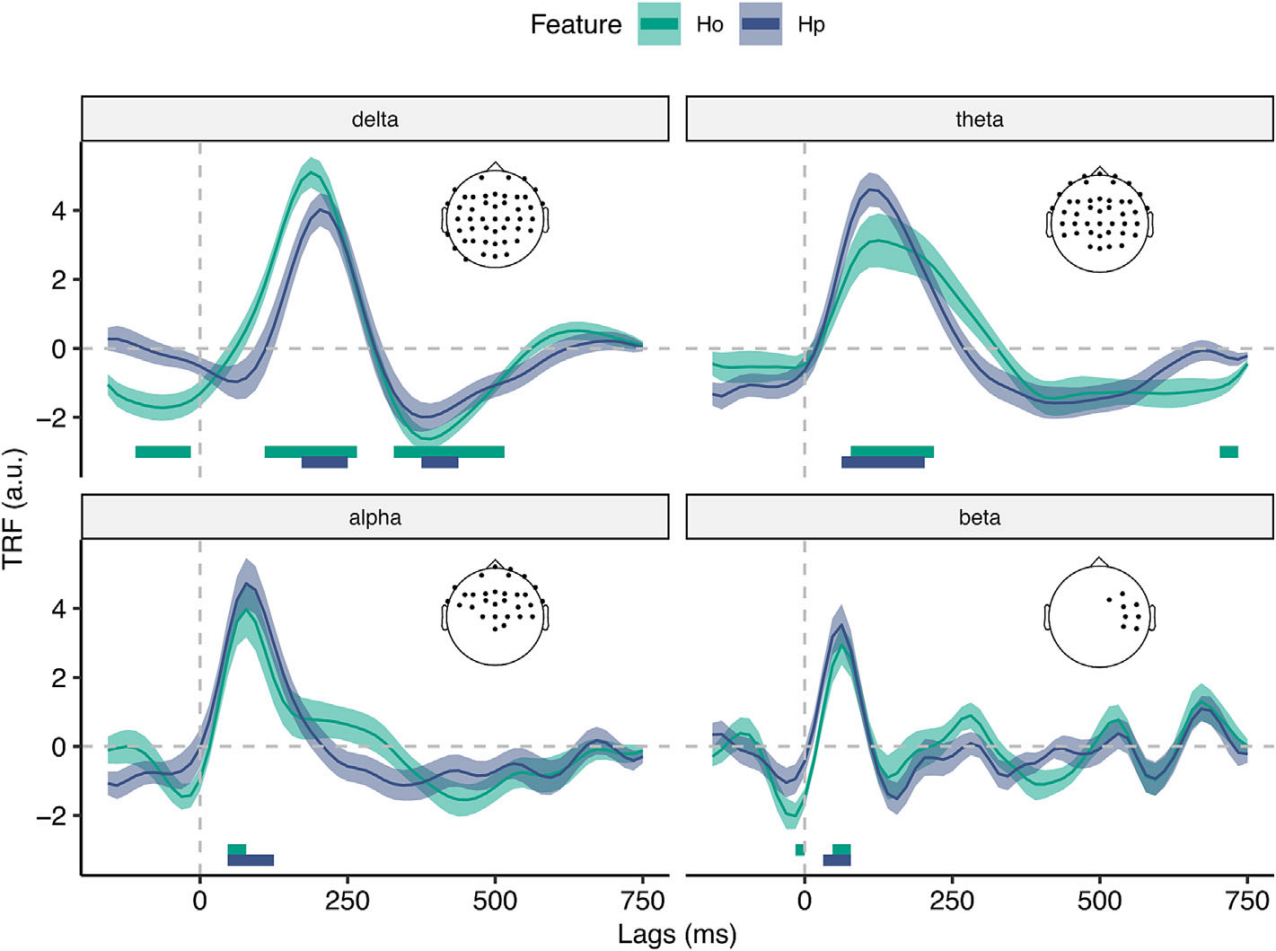
TRF weights. Grand‐average TRF weights across subjects in the selected channels (black dots on the topographies) for the regressors in the AM model that also significantly enhanced EEG reconstruction accuracy. Shaded areas correspond to the standard error of the mean.

To analyse the TRF weights, we selected the electrodes for which AM performed significantly better than A (Figure 2B) and looked for consistent peaks across subjects. As previous results showed that surprisal regressors did not significantly enhance reconstruction accuracy in any frequency band (Figure 2A), their TRF weights would not provide a reliable representation of the encoding. Hence, we limited the TRF weights analysis to the entropy regressors. Here, we found significant peaks in all bands (Figure 3). Before note onset, we found a negative peak of H_o_ in the delta band, from −125 ms to −15 ms, and in the beta band, from −15 ms to 0 ms. After note onset we found early positive peaks in the alpha and beta bands for both features, from 50 to 90 ms; and late positive peaks in the delta and theta bands (delta: from 110 to 250 ms for H_o_ and from 170 to 250 ms for H_p_; theta: from 80 to 220 ms for H_o_ and from 60 to 200 ms for H_p_). At even later latencies, we found a negative peak in the delta band regressors, from 350 to 550 ms. To investigate differences in peak latency between for both regressors within each frequency band, we performed two‐tailed Wilcoxon sign‐rank tests. These tests revealed a significant difference between the peaks of H_p_ and H_o_ in the delta band, from 180 to 220 ms (two‐tailed Wilcoxon sign‐rank test, *Z* = 2.011, *p* = .044, effect size = .450).

### 3.3 Expertise effect

**FIGURE 4:**
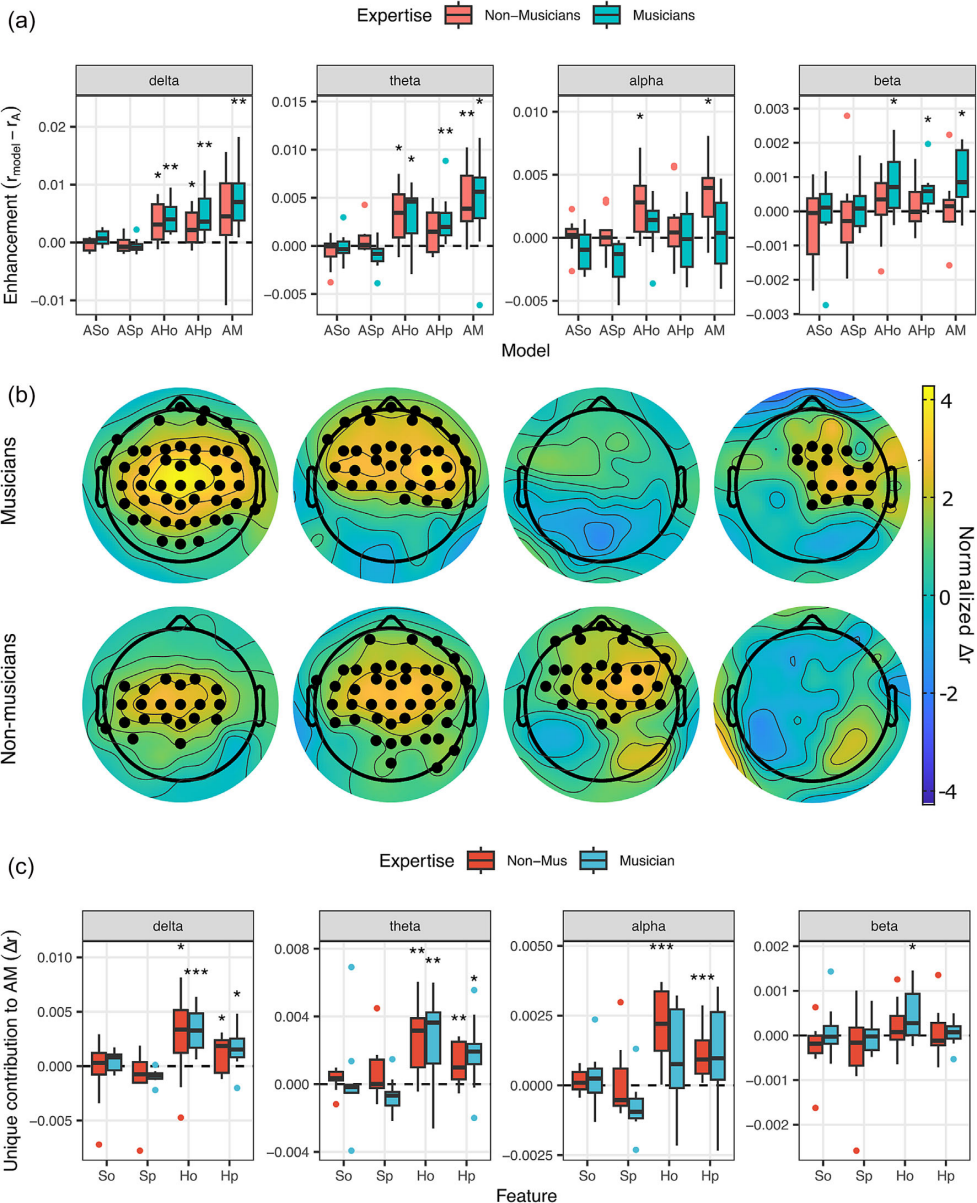
Frequency‐specific EEG reconstruction accuracy enhancement split for musicians and non‐musicians. A, Enhancement in EEG reconstruction accuracy as in Figure 2A (Wilcoxon sign‐rank test; *p < .05, **p < .01, ***p < .001). B, Electrodes with a significant EEG reconstruction accuracy enhancement for the AM model versus the A model (cluster‐based permutation test; *p* < .05, 10,000 permutations). C, Unique contribution of each feature to the EEG reconstruction accuracy of the full AM model (Wilcoxon sign‐rank test; *p < .05, **p < .01, ***p < .001). Coloured dots indicate outlier values.

To investigate the effect of musical expertise on the neural encoding of entropy and surprisal, we conducted all previous analyses separately on the groups of musicians and non‐musicians (Figure 4A). The delta‐ and theta‐band reconstruction accuracy enhancement was consistent with the results on all subjects, with significant effects of AH_o_, AH_p_ and AM in both groups. Yet, AH_o_ showed a significant alpha‐band enhancement for non‐musicians (*p* = .01, effect size = .725), while AH_o_ and AH_p_ enhanced reconstruction accuracy in the beta band for musicians (AH_o_: *p* = .019, effect size = .661; AH_p_: *p* = .003, effect size = .822). Cluster‐based permutation tests comparing the reconstruction accuracy of AM and A models showed an enhancement in the beta band for musicians and in the alpha band for non‐musicians, but not vice versa (Figure 4B). A comparison between the AM and AM‐shuffled models showed a significant enhancement of the AM model for non‐musicians in delta (*p* = .001, effect size = .886), theta (*p* = .002, effect size = .854) and alpha (*p *= .003, effect size = .822), but not in beta (*p *= .188, effect size = .306); and in all bands for musicians (delta: *p* = .001, effect size = .886; theta: *p* = .001, effect size = .886; alpha: *p *= .007, effect size = .757; beta: *p* = .003, effect size = .822). Cluster‐based permutation tests confirmed these effects (*p* < .05). To assess this interaction, we fitted linear mixed effect models with FREQUENCY (alpha, beta), EXPERTISE (musicians, non‐musicians), and their interaction as fixed factors. Model comparison revealed that EXPERTISE did not significantly improve the performance (AIC = −365.62, *χ* ^2^(1) = 2.32, *p* = .128) compared to the null model with only FREQUENCY (AIC = −365.29). Yet, the interaction FREQUENCY x EXPERTISE provided a better fit to the data (AIC = −371.56, *χ* ^2^(1) = 7.94, *p* = .005), as confirmed by an ANOVA test (*F*(1) = 7.91, *p *= .008). Finally, an analysis of the individual contributions by feature (Figure 4C) revealed that musicians had a contribution of H_o_ in the beta band (*p* = .024, effect size = .612), but not in the alpha band (*p *= .065, effect size = .499); while non‐musicians had a contribution of H_o_ (*p* < .001, effect size = .886), and H_p_ (*p *< .001, effect size = .886) in the alpha band, but not in the beta band (H_o_: *p *= .188, effect size = .306; H_p_: *p* =.615, effect size = .081). The results in all other bands were consistent with the results on all subjects.

## 4 DISCUSSION

### 4.1 Feature‐specific contributions to reconstruction accuracy across frequency bands

In the current study, we investigated the neural encoding of prediction uncertainty and prediction errors in music across different frequency bands. We trained several TRF models with different sets of regressors and analysed the EEG reconstruction accuracy. Surprisal and entropy, beyond acoustic features, improved EEG reconstruction accuracy in all frequency bands, except for the gamma band. This result expands on prior findings, as it indicates that these features are encoded in the amplitude modulation of brain rhythms beyond low‐frequencies (Di Liberto, Pelofi, Bianco, et al., 2020; Kern et al., 2022). Furthermore, our work contributes to the broader debate on the role of frequency‐specific neural dynamics in predictive processing, providing evidence for how cognitive processes related to certain predictive components might be associated with neural activity in distinct frequency bands. However, it is important to note that our encoding results cannot determine whether such frequency‐specific brain dynamics reflect intrinsic oscillatory mechanisms as opposed to transient evoked responses, since both may contribute to cortical tracking (Drennan & Lalor, 2019; Obleser & Kayser, 2019). Therefore, our results are to be interpreted in the context of multiple generative frameworks rather than as proof of one theory over another.

A deeper analysis of the feature‐specific neural encoding revealed that entropy, but not surprisal, improved reconstruction accuracy in all frequency bands; likewise, ignoring surprisal regressors did not constitute a significant loss of performance for the model. Linear mixed effect models further confirmed these results, suggesting that entropy improved reconstruction accuracy more than surprisal. Taken together, these findings indicate that entropy, that is the uncertainty of the following notes, plays a key role in music processing and is encoded in the neural response in different frequency bands. However, it is important to note that the lack of evidence for encoding of surprisal is not to be interpreted as evidence of the absence of neural computations related to prediction error. In fact, previous studies found a better encoding of surprisal than entropy in the same music dataset (Di Liberto et al., 2021; Kern et al., 2022) and in naturalistic speech perception experiments (Gillis et al., 2021; Weissbart et al., 2020). This difference might be due to distinct combinations of regressors and different processing mechanisms for speech and music. Moreover, our effect sizes might have been reduced by the use of narrow‐band amplitude modulations rather than power. Yet, our approach was chosen as it is preferable for the interpretation of the TRF weights (Etard & Reichenbach, 2019; Van Canneyt et al., 2021). On the other hand, as we expected surprisal‐related effects to be mostly encoded in the gamma band, it is possible that we did not find evidence for an effect of surprisal because EEG reconstruction accuracy in this frequency band did not show a significant enhancement, even in our omnibus model (AM) trained with all features. High‐frequency activity is difficult to study in EEG using TRFs (Crone et al., 2011; Crosse et al., 2021; Synigal et al., 2020), especially in the current dataset where the sampling frequency of the acoustic variables was limited. In fact, studies using intracranial recordings have found high‐frequency gamma (60–150 Hz) responses linked to prediction errors (Omigie et al., 2019; Sankaran et al., 2024). Hence, different recording methods could mitigate the issue (see for instance Di Liberto, Pelofi, Bianco, et al., 2020; Synigal et al., 2020).

The results of our linear mixed effect models did not provide evidence for a different reconstruction accuracy enhancement based on the type of feature (time, pitch). Yet, we found that the combined regressors for note onset (AM_o_), as opposed to note pitch (AM_p_), produced an enhancement in the reconstruction accuracy that was not limited to low frequencies, rather it extended to high frequencies (Figure S1). This finding seems to suggest that the latter are sensitive to temporal information, as opposed to content. However, we also found that while pitch entropy did not significantly improve the beta‐band reconstruction accuracy in the AM model (Figure 2C), it significantly contributed to the AH model (Figure S2). This result may suggest that the encoding of content predictions may be better when temporal uncertainty is also taken into account. This interpretation is in line with recent work suggesting that “when” and “what” may be more interdependent in the brain's eye view (Ten Oever & Martin, 2024).

Crucially, the present finding that beta‐band activity encodes temporal, but not content, prediction uncertainty does not per se contrast with previous findings that activity in this range is sensitive to content prediction errors (Arnal et al., 2011; Fujioka et al., 2012); neither does it conflict with the proposal that time‐based predictive processing relies on neural dynamics at both high and low frequency (Arnal, 2012). In fact, a prominent framework on predictive processing suggests that beta‐band activity supports temporal predictions by phase‐tuning ongoing low‐frequency neural activity (Morillon & Baillet, 2017; Zalta et al., 2024). In turn, phase‐tuned low‐frequency activity may propagate to sensory regions, where “when” and “what” might be “entangled” via phase coding mechanisms (Arnal et al., 2015; Nelson et al., 2013; Ten Oever et al., 2024; Ten Oever & Sack, 2015). While our results do not necessarily discourage this interpretation that relates predictive processing to oscillatory dynamics, we cannot discard the possibility that our findings could alternatively, or additionally, reflect the involvement of non‐oscillatory mechanisms (Drennan & Lalor, 2019; Obleser & Kayser, 2019).

### 4.2 Frequency‐specific neural dynamics underlying melodic expectations

To further investigate the neural dynamics underlying temporal and content predictions, we analysed the TRF weights of the entropy regressors associated with note onset (H_o_) and pitch (H_p_). In line with prior findings (Di Liberto, Pelofi, Bianco, et al., 2020), we found significant delta‐band peaks for both entropy regressors at around 200 ms after stimulus onset. Additionally, we showed that the delta‐band peak for the H_o_ regressor was significantly earlier than the peak for H_p_. This difference in latency may reflect different processing timescales for temporal and content predictions (Arnal & Giraud, 2012). In line with this interpretation, we found that H_o_ improved reconstruction accuracy in the delta and beta bands before stimulus onset, consistent with prior literature on the joint involvement of delta and beta bands in predictive timing (Arnal et al., 2015; Arnal & Giraud, 2012; Fujioka et al., 2012). While our findings seem consistent with this interpretation, caution should be taken in drawing strong conclusions from these results. In fact, for the beta‐band, there is only a very brief window of significance visible in Figure 3.

Surprisingly, H_o_ significantly improved the reconstruction accuracy also in the theta and alpha bands, with consistent TRF peaks after the stimulus onset. While the role of theta and alpha‐band activity in temporal predictions is still unclear, previous studies have shown alpha‐band desynchronization at the predicted onset time (Rohenkohl & Nobre, 2011; Thut et al., 2006), and theta‐band phase modulation depending on the temporal predictability of the target (Jensen et al., 2012). Additionally, we found a significant contribution of H_p_ in the delta and theta ranges, consistent with prior findings that content uncertainty modulates brain activity in these bands (Donhauser & Baillet, 2020).

The spatial clusters associated with a reconstruction accuracy enhancement in the delta, theta and alpha bands point to a possible involvement of fronto‐central cortical areas, in line with previous literature (Doelling & Poeppel, 2015; Gillis et al., 2021; Quiroga‐Martinez et al., 2020b). On the other hand, the cluster associated with the enhancement in reconstruction accuracy in the beta band seems to be more distributed over the right hemisphere, both at the group level and particularly in musicians (see Figures 2B and 4B). Our control analysis using shuffled melodic information values confirmed this effect only in musicians. Consistently, a plethora of studies have reported the dominance of the right hemisphere in music processing (Bianco et al., 2016; Di Liberto, Pelofi, Bianco, et al., 2020; Koelsch et al., 2007; Oechslin et al., 2018; Sihvonen et al., 2022) and more specifically for temporal predictions during pitch processing (Brodbeck & Simon, 2022; Morillon & Baillet, 2017; Park et al., 2020).

### 4.3 Musical expertise modulates the encoding of melodic expectations

To investigate the influence of musical expertise in the neural encoding of note entropy and surprisal, the performance of TRF models with individual features was contrasted between musicians and non‐musicians. Strikingly, this analysis revealed a significant enhancement in the beta band only for musicians, and in the alpha band only for non‐musicians. Moreover, we confirmed this double dissociation with linear mixed‐effects models, revealing a significant interaction between expertise and frequency band. Previous studies have shown that musical training enhances internal representations of pitch‐related categories (Di Liberto, Pelofi, Shamma, & de Cheveigné, 2020; Hansen et al., 2016; Hansen & Pearce, 2014; Quiroga‐Martinez et al., 2020a) and that beta‐band activity is instrumental to predict note‐timing in musicians (Doelling & Poeppel, 2015). Furthermore, beta‐band modulations have been linked to the (re)activation of internal models (Bressler & Richter, 2015; Spitzer & Haegens, 2017), while alpha‐band activity has been linked to attention or, more generally, to top‐down processes (Haegens et al., 2011; Samaha et al., 2015), which are closely related to prediction. In light of these two views, our finding of an alpha‐beta dissociation might reflect two processing modes depending on musical expertise: musicians might rely on their internal models (i.e., prior musical knowledge), while non‐musicians (who “lack” internal models of music) might process stimuli in an attentive mode. Alternatively, musicians might display a greater alpha‐band desynchronization at note onset relative to non‐musicians (Sorati & Behne, 2019), which may have reduced the alpha‐band power and, potentially, led to a worse fit of the model and lower reconstruction accuracy.

## 5 CONCLUSIONS

The present study advances our understanding of music cognition by revealing new insights into the neural correlates that underlie temporal and content predictions and prediction errors during naturalistic music listening. Our results suggest that: (i) melodic entropy is encoded in the neural response across different frequency bands; (ii) delta‐beta activity encodes temporal uncertainty before stimulus onset and (iii) musical expertise elicits distinct processing modes in the alpha and beta bands. These findings open new avenues for future research into the predictive processing of time and content in the brain.

## AUTHOR CONTRIBUTIONS

**Juan‐Daniel Galeano‐Otálvaro:** Formal analysis (lead); software (lead); visualization (lead); writing—original draft (lead); writing—review and editing (equal). **Jordi Martorell:** Conceptualization (equal); formal analysis (supporting); supervision (supporting); writing—review and editing (equal). **Lars Meyer:** Formal analysis (supporting); funding acquisition (lead); writing—review and editing (equal). **Lorenzo Titone:** Conceptualization (equal); formal analysis (supporting); project administration (lead); supervision (lead); writing—original draft (supporting); writing—review and editing (equal).

## CONFLICT OF INTEREST STATEMENT

The authors declare no competing interests.

### PEER REVIEW

The peer review history for this article is available at https://www.webofscience.com/api/gateway/wos/peer‐review/10.1111/ejn.16581.

## Supporting information


**Figure S1.** Enhancement in EEG reconstruction accuracy for models trained with additional features versus the baseline model trained with only acoustic features. AM_o_ and AM_p_ are models trained with both entropy and surprisal metrics for note onset and pitch, respectively (*p < .05, **p < .01, ***p < .001; Wilcoxon sign‐rank test). Black dots indicate outlier values.

**Figure S2.** Unique contribution of each entropy regressor to the AH model (*p < .05, **p < .01, ***p < .001; Wilcoxon sign‐rank test).

## Data Availability

The dataset on which this study is based was obtained from Di Liberto et al. (2021) and is openly available at https://doi.org/10.5061/dryad.g1jwstqmh.
